# Mitigating IL-12-induced toxicities: A path forward with TNF inhibition

**DOI:** 10.1016/j.omton.2025.200951

**Published:** 2025-03-04

**Authors:** Julia Davydova, Christopher J. LaRocca

**Affiliations:** 1 Department of Surgery, University of Minnesota, 420 Delaware Street SE, MMC 195, Minneapolis, MN 55455, USA; 2 Masonic Cancer Center, University of Minnesota, Minneapolis, MN, USA; 3 Institute of Molecular Virology, University of Minnesota, Minneapolis, MN, USA

## Main text

The manuscript by the Bartee group addresses critical challenges in the optimization of interleukin (IL)-12-mediated cancer immunotherapy, offering potential strategies to improve its safety and efficacy.^1^ The study investigates a recombinant oncolytic myxoma virus expressing a soluble PD1 and IL-12 fusion protein with the goal of mitigating the well-documented toxicities associated with IL-12 treatment. The authors provide valuable insights into the mechanisms underlying IL-12-induced toxicities and propose novel approaches to overcome these limitations.

IL-12 is a potent cytokine with remarkable anti-tumor properties. By activating both innate (natural killer cells) and adaptive (cytotoxic T lymphocytes) immune responses, IL-12 effectively bridges the two arms of immunity. Additionally, IL-12 exhibits anti-angiogenic properties, further enhancing its potential as an anti-cancer agent. However, clinical translation of IL-12 therapy has been hindered by severe systemic toxicities observed in early clinical trials, including neutropenia, thrombocytopenia, and hepatic dysfunction.^2^^,^^3^ These toxicities have necessitated the development of localized delivery strategies, including the use of oncolytic viruses engineered to express IL-12 within the tumor microenvironment.^4^

A major highlight of this study is the identification of tumor necrosis factor alpha (TNF-α) as a key mediator of IL-12-induced toxicities. The authors demonstrate that the systemic effects of IL-12 treatments are dependent on TNF expression, a conclusion consistent with prior studies. Further, the authors suggest that these toxicities can be mitigated using the clinically available TNF blocker etanercept (Enbrel). By neutralizing TNF, etanercept not only reduces toxicities, but also enhances intratumoral T cell viability, thereby improving the therapeutic efficacy of the IL-12-expressing oncolytic virus. This dual benefit of mitigating adverse effects while boosting anti-tumor immunity highlights the potential of TNF blockade as a valuable adjunct to IL-12-based therapies.

The study further emphasizes the need for further research to optimize TNF-blocking strategies in conjunction with IL-12 therapies. With five FDA-approved TNF inhibitors available (each with distinct specificities and mechanisms of action),^5^ it is crucial to identify the most effective agent for combination therapy with oncolytic viruses. For example, etanercept preferentially targets membrane-bound TNF, a property that may enhance its efficacy in the tumor microenvironment.

While this study provides valuable insights into IL-12-mediated immunotherapy, certain aspects warrant further investigation. A more comprehensive evaluation of IL-12-induced toxicities, along with a detailed assessment of the overall safety profile of the oncolytic myxoma virus as a delivery vector, would further strengthen the findings. Additionally, exploring whether TNF-blocking antibodies influence the therapeutic efficacy of the viral vector could provide further clarity on the potential of such combination regimens. Future studies may also help determine if alternative TNF inhibitors offer advantages over etanercept in this context. However, it is important to recognize the challenges in preclinical research, as many TNF inhibitors exhibit limited efficacy in murine models, which can complicate the translation of findings into clinical applications.^6^

In conclusion, this study advances our understanding of the role of TNF in IL-12-induced toxicities and highlights the therapeutic potential of TNF blockade in enhancing IL-12-based therapies. By integrating TNF inhibitors into immunotherapeutic regimens, this approach could broaden applications in oncology and improve patient outcomes. Furthermore, the observed benefits of TNF blockade are not restricted to IL-12-based therapies and could extend to other immunotherapies, such as immune checkpoint inhibitors. These observations underscore the potential of TNF inhibitors as a transformative tool in the ongoing evolution of cancer immunotherapy.

## Acknowledgments

The authors would like to acknowledge the funding from the 10.13039/100007249University of Minnesota Masonic Cancer Center, the Randy Shaver Community Cancer Fund, the Minnesota Colorectal Cancer Research Foundation, NIH/10.13039/100006108NCATS (KL2TR002492, K12TR004373, and 1UM1TR004405), and NIH/10.13039/100000054NCI R01 CA276179.

## Declaration of interests

The authors have no relevant disclosures to report.
